# A hypertensive patient with multiple intracerebral hemorrhages due to brain metastases

**Published:** 2009-11-25

**Authors:** A Ghitoiu, CM Rusu, D Slăvoaică, E Aigyul, BO Popescu

**Affiliations:** * Department of Neurology, University Hospital, Bucharest Romania; ** ‘Carol Davilaߣ University of Medicine and Pharmacy, BucharestRomania

**Keywords:** brain hemorrhage, acute onset, brain metastases

## Abstract

We report the case of a 55–year–old man, hypertensive, who presented to the Emergency Room with intense occipital cephaleea, nausea, vomiting and disturbance of balance. The peculiarity of this case was given by the simultaneous presence of two brain hemorrhagic lesions and an unusual hypodensity with digitiform borders at cerebral CT scan, which suggested a different  etiology than hypertension and leaded us to further investigations, which confirmed the diagnosis of lung cancer with multiple brain metastases.

## Background

The most common cause of intracerebral hemorrhage in middle aged patients is chronic uncontrolled hypertension. Other causes are rupture of an aneurysm or an arteriovenous malformation, arteriopathy, anticoagulant therapy, cerebral venous thrombosis, hemorrhagic transformation of an ischemic infarct, trauma, sympathomimetic drug use and brain tumor (1). Brain metastases are the most frequent cerebral tumors in adults, lung cancer being the leading primary source. About 80% of the metastases are located in the cerebral hemispheres and 20% in the posterior fossa structures. Twenty five percent of the brain metastases have an intratumoral hemorrhage as the cause of first clinical signs [2].

Intracerebral hemorrhage can occur in any part of the brain. The bleeding might be located to one hemisphere (lobar intracerebral hemorrhage) or to other brain structures. Predilection sites for intracerebral hemorrhage due to hypertension include: basal ganglia (40–50%), thalamus (10–15%), pons (5–12%) and cerebellum (5–10%). Chronic hypertension generates a small vessel vasculopathy, in time, characterized by lipohyalinosis, fibrinoid necrosis and development of Charcot–Bouchard aneurysms affecting and penetrating arteries throughout the brain [1].

## Case Report

We report here the case of a 55–year–old male, patient, smoker (20 cigarettes/day for 30 years), hypertensive (highest values of 210/120 mmHg, diagnosed with hypertension at the age of 46), with intermittent use of antihypertensive treatment at home, who presented at the hospital with abrupt onset of intense occipital headache, nausea, vomiting and disturbance of balance. The physical examination revealed a conscious and cooperative patient with a high value of arterial blood pressure (175/80mmHg), a cardiac sinus rhythm of 84/min, astasia–abasia, left superior limb ataxia, positive bilateral Babinski sign. He had no other personal medical history but a family history of cerebrovascular diseases and cancer (two uncles: one who died after an intracerebral hemorrhage and the other one due to brain metastasis from primary urinary bladder tumor). The complete blood cell count was normal, the biochemical blood tests revealed high glucose levels (213mg/dL), slight increase of transaminases (GOT= 45 U/L, GPT= 58U/L), dyslipidemia with hypercholesterolemia and hypertriglyceridemia (cholesterol=275mg/dL, triglyceride= 170mg/dL), increased fibrinogen (463mg/dL) and high sedimentation rate (33mm/h), spontaneous INR of 1.24 and PT =13.6/s.

**Fig 1:**
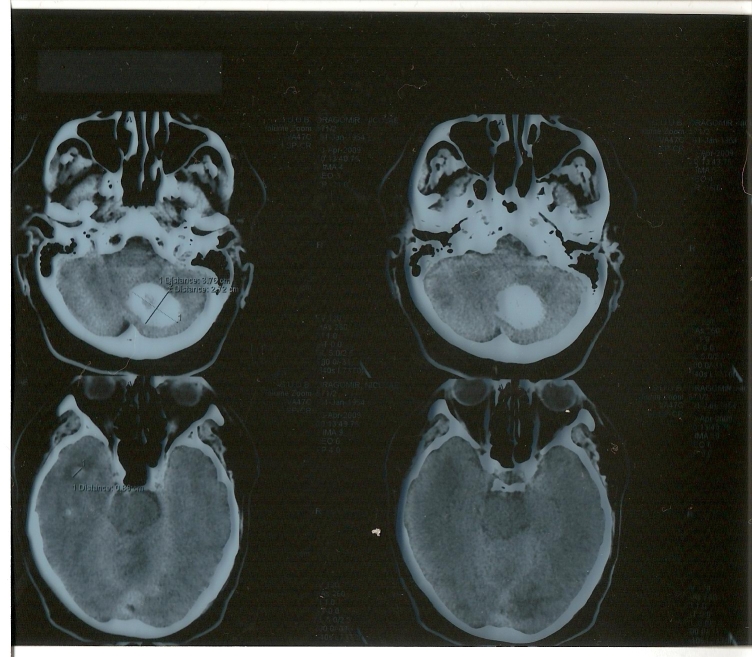
The brain CT scan without contrast performed in the admission day revealed a left cerebellar hemorrhage with surrounding edema and compressive effect in the fourth ventricle but no appreciable underlying mass. A small hemorrhage of 9 mm with perilesional edema localized in the right temporal lobe was also identified.  A left occipital hypodense brain area with digitiform borders was found as well (white arrow).

**Fig 2:**
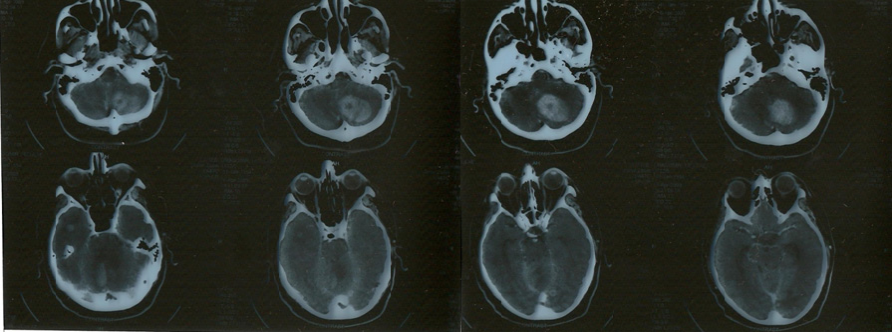
The brain contrast CT scan performed 24 hours after admission revealed multiple zones with contrast enhancement localized in both cerebellar hemispheres, in the right temporal lobe, in the right occipital lobe and in the left occipital lobe, one of them associated with an acute hemorrhage (a left cerebellar hematoma). The aspect of the lesions was suggestive for cerebral metastases.

The CT cerebral scan performed at the admission revealed a left cerebellar hemorrhage (3.7/2.7cm) with perilesional edema with compressive effect on the fourth ventricle and minimal efraction at this site, but no appreciable underlying mass and a small hemorrhage of 9mm localized in the right temporal lobe with minimal perilesional edema without ventricular efraction (Fig 1). A left occipital hypodense brain area with digitiform borders was also found (Fig 1, white arrow). Due to these initial unusual findings, the next step was to perform a brain CT with contrast (Fig 2), which revealed multiple zones with post–contrast enhancement localized in both cerebellar hemispheres (of 17/16 mm in the right cerebellar hemisphere and of 24/25mm in the left cerebellar hemisphere), in the right temporal lobe (9mm), in the right occipital lobe (7mm) and in the left occipital lobe (1cm) with surrounding edema, one of them associated with an acute hemorrhage (a left cerebellar hematoma). The aspect of the lesions was suggestive for cerebral metastases. This finding led us to standard further investigations appropriate in such a clinical situation. A chest X–ray was performed and revealed multiple opacities with irregular borders and the patient was eventually diagnosed with lung cancer after bronchoscopy. The patient received depletive (Manitol), corticoid (Dexamethasone), antihypertensive and pain therapy until being evaluated by the oncology service.

## Discussion

Although the patient had multiple vascular risk factors (hypertension improperly treated, dyslipidemia, and smoking) and, at his age, the risk for cerebral bleeding as a complication of the vascular disease was high, a careful examination of the brain CT scan suggested that the hemorrhage was due to a less probable cause, namely multiple brain metastases of lung cancer origin. Moreover, from the clinical point of view, there was a discrepancy between the massive lesions revealed by the CT scan and the minor clinical signs, which made the pure vascular character of the disease improbable. In contrast to the cerebrovascular disease, space occupying lesions which develop over a long time do not generate dramatic neurological symptoms. However, multiple simultaneous intracerebral hemorrhages (SIHs) occur rarely, in a series of hemorrhagic strokes only 2.8% of patients being diagnosed with SIHs [3]. In different studies, SIHs were associated with cerebral amyloid angiopathy, vasculitis, hematologic disorders, illicit drug use, anticoagulant therapy, or with hemorrhagic transformation of multiple cerebral infarcts [4, 5]. The case presented in this report illustrates the caution needed for the correct diagnosis of SIHs.
